# Optimization of the isolation procedure and culturing conditions for hepatic stellate cells obtained from mouse

**DOI:** 10.1042/BSR20202514

**Published:** 2021-01-22

**Authors:** Thanh Minh Dang, Trinh Van Le, Huy Quang Do, Van Thuan Nguyen, Ai Xuan Le Holterman, Loan Tung Thi Dang, Nhan Chinh Lu Phan, Phuc Van Pham, Son Nghia Hoang, Long Thanh Le, Gabriele Grassi, Nhung Hai Truong

**Affiliations:** 1 Laboratory of Stem Cell Research and Application, University of Science, VNUHCM, Ho Chi Minh City, Vietnam; 2 University of Science, Viet Nam National University, Ho Chi Minh City, Vietnam; 3 School of Biotechnology, International University, VNUHCM, Ho Chi Minh City, Vietnam; 4 Department of Pediatrics and Surgery, University of Illinois College of Medicine, Chicago and Peoria, Illinois, U.S.A; 5 Faculty of Biology and Biotechnology, University of Science, VNUHCM, Ho Chi Minh City, Vietnam; 6 Stem Cell Institute, University of Science, VNUHCM, Ho Chi Minh City, Vietnam; 7 Animal Biotechnology Department, Institute of Tropical Biology, Vietnam Academy of Science and Technology, Ho Chi Minh City, Vietnam; 8 Department of Life Sciences, Cattinara University Hospital, Trieste University, Trieste, Italy

**Keywords:** Hepatic Stellate Cells, HSCs activation, liver fibrosis, Nycodenz, Quiescent stellate cells

## Abstract

Liver fibrosis (LF) mortality rate is approximately 2 million per year. Irrespective of the etiology of LF, a key element in its development is the transition of hepatic stellate cells (HSCs) from a quiescent phenotype to a myofibroblast-like cell with the production of fibrotic proteins. It is necessary to define optimal isolation and culturing conditions for good HSCs yield and proper phenotype preservation for studying the activation of HSCs *in vitro*. In the present study, the optimal conditions of HSC isolation and culture were examined to maintain the HSC’s undifferentiated phenotype. HSCs were isolated from Balb/c mice liver using Nycodenz, 8, 9.6, and 11%. The efficiency of the isolation procedure was evaluated by cell counting and purity determination by flow cytometry. Quiescent HSCs were cultured in test media supplemented with different combinations of fetal bovine serum (FBS), glutamine (GLN), vitamin A (vitA), insulin, and glucose. The cells were assessed at days 3 and 7 of culture by evaluating the morphology, proliferation using cell counting kit-8, lipid storage using Oil Red O (ORO) staining, expression of a-smooth muscle actin, collagen I, and lecithin-retinol acyltransferase by qRT-PCR and immunocytochemistry (ICC). The results showed that Nycodenz, at 9.6%, yielded the best purity and quantity of HSCs. Maintenance of HSC undifferentiated phenotype was achieved optimizing culturing conditions (serum-free Dulbecco’s Modified Eagle’s Medium (DMEM) supplemented with glucose (100 mg/dl), GLN (0.5 mM), vitA (100 μM), and insulin (50 ng/ml)) with a certain degree of proliferation allowing their perpetuation in culture. In conclusion, we have defined optimal conditions for HSCs isolation and culture.

## Introduction

Hepatic stellate cells (HSCs) are liver-specific mesenchymal cells that reside within the subendothelial space of Disse around the liver sinusoid [1–3]. In healthy conditions, HSCs remain in the quiescent form and function primarily as fat-storing cell representing the largest reservoir of vitamin A (vitA) in the body [4]. In their quiescent state, HSCs express Glial fibrillary acidic protein (GFAP) [5–8] and desmin [9,10]. Following toxicity-induced or viral-induced liver injury, HSCs undergo activation to a myofibroblast-like cell [11] characterized by the loss of vitA-containing lipid droplets (LDs) and by increased expression of many fibrogenic proteins including collagen type 1 α1 (Col1a1), α-smooth muscle actin *(α-SMA*), all considered to be primary markers of the stellate cell activation [4,12–14]. Activated HSCs have become recognized as the primary cell contributor to hepatic fibrosis [15]. A precise characterization of HSC’s features together with the determination of the underlying mechanisms responsible for their transdifferentiation, is a prerequisite for the development of novel therapeutic intervention to liver fibrosis (LF).

HSCs isolation and culture are valuable tools to study the HSCs transdifferentiation process. Density gradient centrifugation has been considered the ‘gold standard’ to separate HSCs with high isolation efficiency [16–21]. Many current studies have used various gradient substances such as Stractan [22,23], Percoll [24–26], Optiprep [27–30], Metrizamide [31], but the most popular is Nycodenz. However, the yield and purity of the isolated HSCs varies greatly among the published works mainly due to the wide range of Nycodenz concentrations used.

Beyond defining the optimal isolation procedure, it is also important to find culturing conditions to maintain HSCs in the quiescent phenotype. This is not a trivial technical aspect as HSCs acquire the activated phenotype typically occurring in LF within a few days after isolation and culture [11,15,32,33]. This rapid switch to the activated phenotype represents an obstacle to study HSCs physiology *in vitro*. Therefore, many studies have investigated the possibility of maintaining the HSC quiescent phenotype *in vitro* by modulating the culture conditions. In this regard, vitA and insulin have been reported to maintain HSC in the quiescent state [34]; glucose and l-glutamine (GLN) affect both growth and activation of HSCs [35–40]. Therefore, the appropriate combination of these elements in culture could potentially keep HSCs quiescent while promoting proliferation.

The present study was undertaken to evaluate the optimal Nycodenz concentration for isolating HSCs with high purity and yield. We also investigated the culture medium composition to maintain the quiescent phenotype while retaining the ability to proliferate.

## Methods

### Animals

Four- to five-months old BALB/c mice (Pasteur Institute, HCM city, Vietnam) were kept in the Microventilation cage system (THREE-SHINE Inc., Korea), 12-h dark/light cycle, and were fed with regular chow *ad libitum*. All *in vivo* experiments were performed at Laboratory of Animal Care and Use (Stem Cell Institute, VNU-HCM- University of Science, Vietnam) following the guidelines of the EU directive (2010/63/EU) and the permission from the Animal Ethics Committee of the Stem Cell Institute, VNU-HCM- University of Science, Vietnam (Ref. No.: 200501/SCI-AEC).

### Chemicals and reagents

The basic medium consisted of Dulbecco’s Modified Eagle’s Medium—DMEM with low glucose concentration (100 mg/dl), GLN-free; other supplements: fetal bovine serum—FBS and glucose solution, GlutaMAX™, purchased from Gibco, U.S.A. The primary antibodies used in Flow cytometry, immunocytochemistry (ICC) staining, and Western blot were anti-desmin (ab8592), anti-SMA (ab15734), anti-GFAP (ab68428), anti-collagen I (ab21286), anti-glyceraldehyde-3-phosphate dehydrogenase (GAPDH) (ab181602). The secondary antibody was Alexa Fluor 488-conjugated (ab150077) and Goat Anti-Rabbit IgG H&L (HRP) (ab6721). All antibodies were purchased from Abcam, U.S.A. The antibodies were diluted in antibody diluent, consisting of Tris-buffered saline, 0.1% Tween 20 (TBST) solution, 1% bovine serum albumin—BSA. The blocking buffer was prepared from TBST, 4% goat serum (Gibco, Massachusetts, U.S.A), 1% BSA. The permeabilization solution contained PBS and 0.1% Triton X-100. Oil Red O (ORO), VitA, BSA, and insulin were acquired from Sigma–Aldrich, U.S.A.

### HSCs isolation

**Figure 1:**
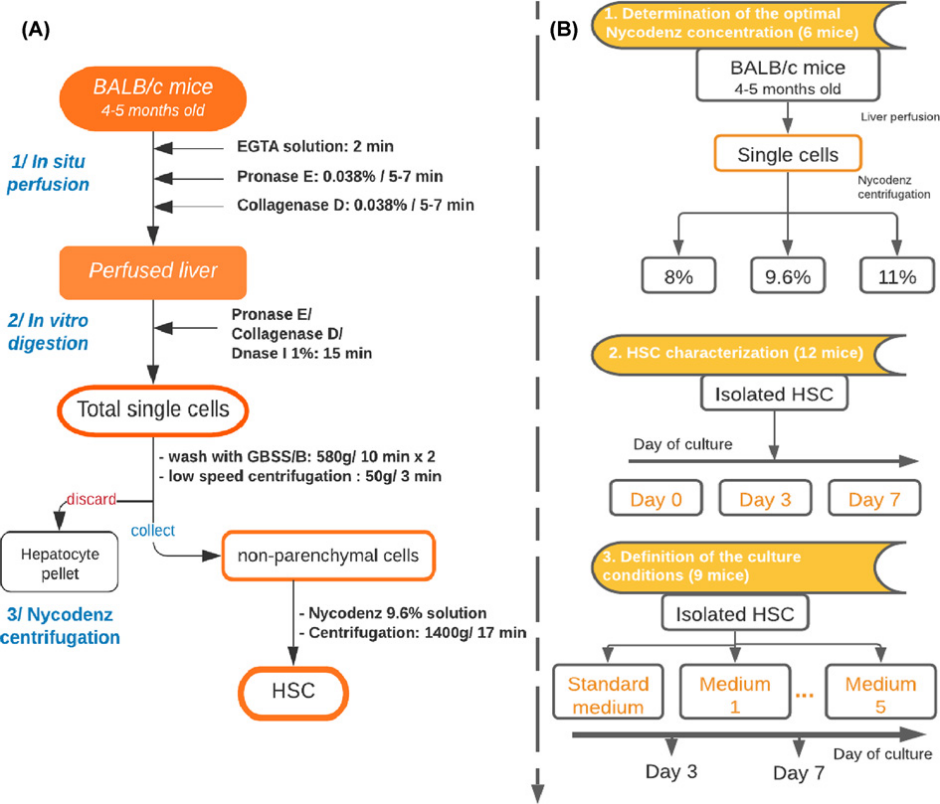
The flowchart of study design (**A**) Flow chart of the isolation procedure of HSCs from mouse livers and (**B**) Experimental design of HSCs culture.

HSCs were isolated from BALB/c mice following the protocol published in 2015 [18] with some modifications (Figure 1A). Briefly, mouse was given intramuscular injections of 20 mg/kg of Ilium xylazil-20 (Troy Laboratories, Australia) and 14 mg/kg of Zoletil (Virbac, France) to induce deep anesthesia. Then, livers were digested by *in situ* perfusion with EGTA solution (Sigma–Aldrich, U.S.A.) for 2 min, pronase E (Merck, Germany), and collagenase D 0.038% for 5–7 min each (Roche Diagnostics, Germany). The mice were killed due to the change in circulation and the opening of diaphragm for liver perfusion. Next, the liver was excised into small pieces and further digested with pronase E and collagenase D supplemented with DNase I 1% (Roche Diagnostics, Germany) *in vitro* for 15 min. The digested solutions were then filtered through a 70-μm cell strainer and subjected to low-speed centrifugation (50×***g*** for 3 min) to discard pelleted hepatocytes. The single-cell suspension was divided equally into three 15-ml tubes, mixed with a solution of Nycodenz (Axis-Shield, U.K.) to reach the final concentrations of 8, 9.6, and 11% separately and centrifuged at 1400×***g*** for 20 min. HSCs were collected from the white layer. The number of isolated cells and cell viability were determined by Trypan Blue staining (Sigma–Aldrich, U.S.A.).

### Purity analysis

The isolated cells were fixed with 4% paraformaldehyde (PFA) for at least 15 min, washed with PBS twice, permeabilized for 15 min, incubated with blocking buffer for 30 min at room temperature (RT) with shaking. Afterward, the cells were incubated with primary antibody anti-desmin (1:200) for 1 h at RT, followed by washing twice with PBS, 5 min each. Then, the secondary antibody (1:500) was added and incubated at RT for 1 h; the unbinding antibody was eliminated by washing twice with PBS, 5 min each. The percentage of desmin-positive cells was then analyzed using the FACS Calibur flow cytometer (BD Biosciences, U.S.A.).

### Culturing of HSCs

HSCs were cultured on plastic dishes coated with fibronectin. Quiescent HSCs were cultured in standard medium for 1 day. Then, the standard medium was replaced with the different test media reported in Table 1. The cells were assessed at days 3 and 7 after adding the test medium (Figure 1B).

**Table 1:** Combination of supplements in test culture media

Medium ID	FBS (10%)	VitA (100 μM) + insulin (50 ng/ml)	GLN (0.5 mM)	Glucose concentration (mg/dl)	
				100	450
**Standard**	+	-	+		+
**Med 1**	-	-	+		+
**Med 2**	-	+	-	+	
**Med 3**	-	+	-		+
**Med 4**	-	+	+	+	
**Med 5**	-	+	+		+

### LD staining and quantification

Briefly, the cultured cells were fixed with 4% PFA. The stock ORO 0.5% was prepared in absolute isopropanol. Before using, the stock ORO was diluted 3:2 in distilled water and left for 10 min at RT to obtain the final working solution. Cells were stained with the working solution for 20 min at RT, followed by a three-time rinse with PBS. The nuclei were counterstained with Hematoxylin for 1 min before light microscope observation. LDs area was measured by ImageJ software (NIH, U.S.A.) that quantify the red-stained area in the image as decribed in ImageJ User Guide. A minimum of 300 cells per well (10%) were used for analyzing LDs.

### Gene expression examination by qRT-PCR

Total RNA was extracted using column-based (with on-column Dnase treatment) total RNA Purification Kit (Norgen Biotek, Canada). The concentration of isolated RNA was measured using a spectrophotometer (Eppendorf, Germany). Then, 1 μg of RNA was reverse transcribed into cDNA using SensiFAST™cDNA Synthesis Kit (Bioline, U.K.). Subsequently, cDNA was employed as template for quantitative-PCR using SensiFAST™SYBR®Hi-ROX Kit (Bioline, U.K.) on the Light Cycler-480 Instrument-II (Roche, Switzerland). The primer sequences used in the present study are shown in Table 2. The expression level of the target genes was normalized to the housekeeping gene (*Gapdh*) and analyzed using the Livak method (2^−ΔΔ*C*_t_^).

**Table 2:** Sequences of primers

Gene	Forward (5′–3′)	Reverse (5′–3′)	ID
***Gapdh***	AAGTTGTCATGGATGACC	TCACCATCTTCCAGGAGC	NM_008084.3
α***-SMA***	GCATCCACGAAACCACCTA	CACGAGTAACAAATCAAAGC	NM_007392.3
***Collagen I***	CAATGGCACGGCTGTGTGCG	AGCACTCGCCCTCCCGTCTT	NM_007742.4
***Lrat***	CTGACCAATGACAAGGAACGCACTC	CTAATCCCAAGACAGCCGAAGCAAGAC	NM_023624.4
***Pecam1***	AGTCAGAGTCTTCCTTGCC	AGTTCAGAAGTGGAGCAGCT	NM_001032378.2
***Clec4f***	CTTCGGGGAAGCAACAACTC	CAAGCAACTGCACCAGAGAAC	NM_016751.3

### ICC staining

The adherent cells were rinsed with PBS twice and fixed with 4% PFA. Then, the cells were permeabilized, incubated with blocking buffer for 30 min. Afterward, the cells were incubated with primary antibody against desmin (1:200), GFAP (1:100), or α-SMA (1:300) overnight at 4°C followed by washing two times, 5 min each. Then the primary antibodies were detected by incubating with secondary antibody Alexa Fluor 488-conjugated (1:500) for 1 h at RT. The nuclei were stained with DAPI (1:5, Santa Cruz, U.S.A.), for 15 min. The fluorescent images were acquired by the Cytell™ Cell Imaging System (GE-Healthcare, U.K.).

### Proliferation assay

The cell count was quantified using Cell Counting Kit 8–CCK8 assay. At the assessment time point, the medium was replaced with 100 μl of fresh medium followed by the addition of 10 μl of CCK8 Kit reagent. The cells were then incubated in the incubator for 4 h before the absorbance at 450 nm was measured using DTX-880 microplate reader (Beckman Coulter, U.S.A.). The absorbance of a series of cell concentrations was measured to serve as the standard curve.

### Cell cycle analysis

Cultured HSCs were fixed with 4% PFA. The cells were stained with DNA-binding dye DAPI (Sigma–Aldrich, U.S.A.). All stages of the cell cycle were identified based on a complete single-color assay workflow of the Cytell™ Cell Imaging System (GE-Healthcare, U.K.).

### Western blot

The cells were lysed with ice-cold RIPA buffer (ab156034) for 10 min. The lysate then was centrifuged at 13000 rpm for 10 min at 4°C, the supernatant was collected. The total protein concentration was determined using BCA protein assay kit (ab102536). Ten micrograms of protein samples was mixed with LDS Sample Buffer (ab119196) and heated for 10 min at 70°C. The protein samples were loaded on to SDS/PAGE gel (ab139596) with equal amounts. Gel running was performed with Running buffer (ab119197) at 50 V for 2 h. Protein samples were transferred to PVDF membrane (ab133411) at 90 V for 1.5 h. The membrane was treated with Blocking buffer (ab126587) for 1 h at RT with shaking. The membrane was incubated with primary antibody anti-SMA (1:2000), anti-collagen I (1:5000) overnight at 4°C. Anti-GAPDH antibody (1:10000) was used as the control. The membrane was washed three times with TBST, 5 min each before incubated with secondary antibody Goat Anti-Rabbit IgG (HRP) (1:10000) at RT for 1 h with shaking. ECL Kit (ab65623) was used to detect the blots and the imaging was conducted with X-ray film. All the reagents were purchased from Abcam, U.S.A.

### Statistical analysis

All experiments were performed in triplicate. GraphPad Prism software was used for statistical analysis. All data were shown as mean ± standard error of the mean (SEM). Statistical differences were assessed by One-way analysis (ANOVA) or Student’s *t* test (*P*-value <0.05 was considered statically different), depending on the number of groups.

## Results

### Determination of the optimal Nycodenz concentration for HSCs isolation

**Figure 2:**
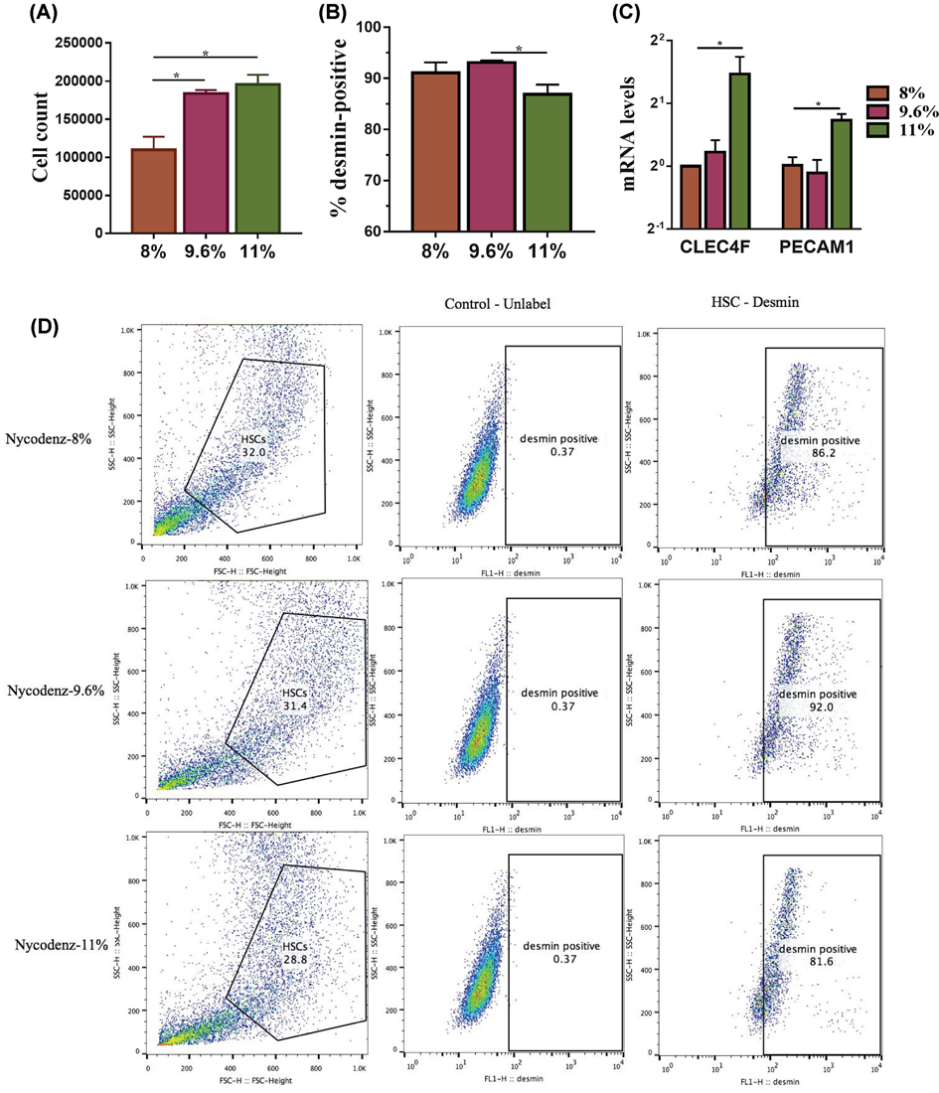
Evaluation of different Nycodenz concentrations on HSCs purification The cell yield using Nycodenz concentrations at 8, 9.6, and 11% was evaluated by cell counting (**A**). Enrichment in HSCs cells was determined by cytofluorimetry (**D**) for desmin-positive cells as quantified in (**B**). The amount of contaminating cells (Kupffer and endothelial cells) was determined measuring the mRNA expression levels of *clec4f* and *pecam1* genes, as markers of Kupffer and endothelial cells (**C**) after normalizing to the housekeeping gene *Gapdh*. Data are shown as means ± SEM, *n*=3, *P<0.05. Abbreviations: *clec4f*, C-type lectin domain family 4 member F; *pecam1*, platelet endothelial cell adhesion molecule-1.

The amount of cells isolated using Nycodenz 11% was not different from that of Nycodenz 9.6% being higher than with Nycodenz 8% (Figure 2A). The purity data showed a significant lower (*P*-value <0.05) proportion of desmin-positive cells using the Nycodenz concentration of 11% compared with 8 and 9.6% (Figure 2B,D).

The mRNA levels of *clec4f* (C-type lectin domain family 4 member F) (marker of Kupffer cells) and platelet endothelial cell adhesion molecule-1 (*pecam1*) (marker of endothelial cells) (*P*-value <0.05, Figure 2C) in cells isolated from Nycodenz 11% were found to be remarkably higher than that from Nycodenz 9.6 and 8%. Based on the above results (amount of isolated cells and purity), cells isolated by Nycodenz 9.6% were considered for further testing.

### Cell count and viability of HSCs from the isolation procedure

**Figure 3:**
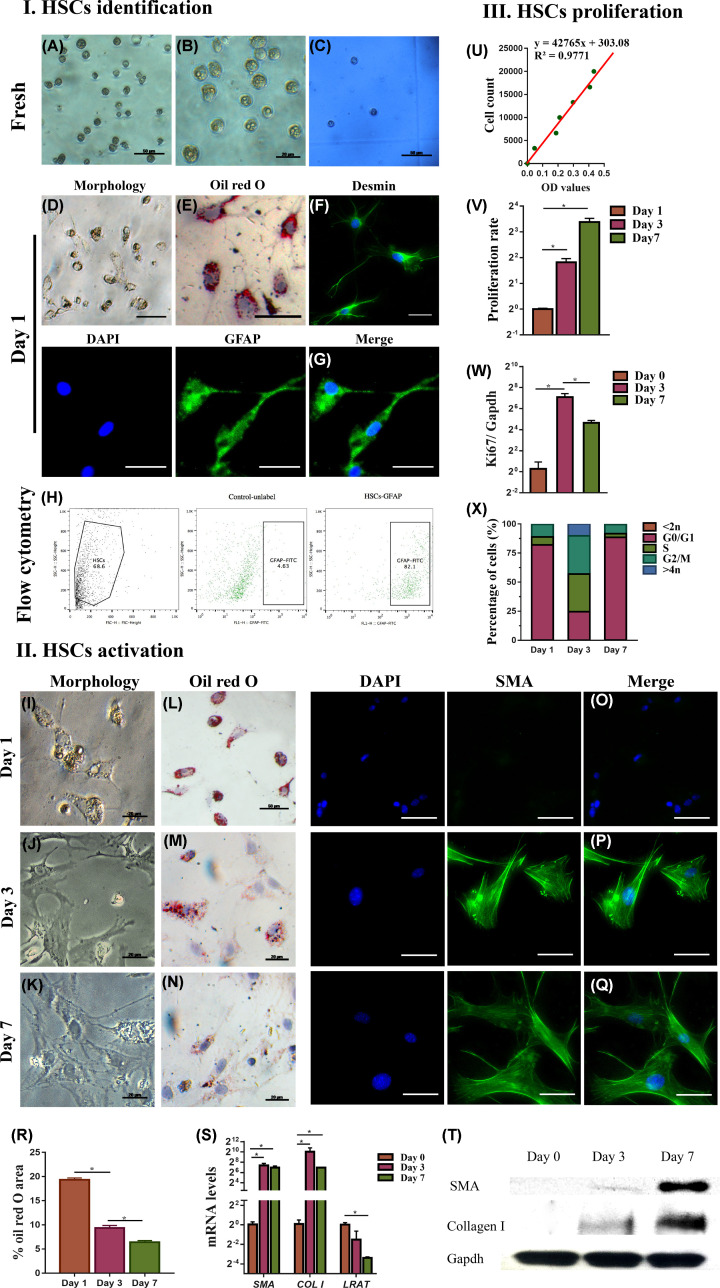
HSCs characterization Freshly isolated cells (**I**) and cells isolated at days 1 and 7 (**II**) were evaluated by phase-contrast microscopy (**A,B,D,I**–**K**), by ORO dye staining for LDs (**E,L**–**N,R**), by hemocytometer and Trypan Blue staining for viability (**C**), by flow cytometry for GFAP marker (**H**), by desmin immunofluorescence (**F**), GFAP (**G**) and a-SMA (**O–Q**) and mRNA levels of a-SMA, COL I and LRAT (**S**), western blot for a-SMA and collagen I (**T**). (**III**) HSCs proliferation evaluated by cell counting (**U**), by proliferation rate with CCK8 assay (**V**), by ki67 mRNA levels normalized to the housekeeping gene *Gapdh* (**W**), by cell cycle analysis (**X**). Data are shown as means ± SEM, *n*=3, *P<0.05, scale bar: 50 μm were not noted. Abbreviation: COL I, type I collagen.

With Nycodenz 9.6%, we could recover 1.96 ± 0.07 million HSCs per mouse. The viability of the isolated cells was higher than 95% assessed by a hemocytometer (Figure 3I(C)).

### HSCs characterization

#### Morphology of HSCs

Bright-field microscope images of freshly isolated HSCs showed the irregular shape and the irregular surface from multiple cytoplasmic LDs (Figure 3I(A)). Under phase-contrast microscope, the retinoid-containing LDs were shining as yellowish-orange color (Figure 3I(B)). The flow cytometry analysis showed more than 82% of cells were positive for GFAP, marker of the quiescent phenotype (Figure 3I(H)). Twenty-four hours after isolation, staining with ORO dye showed LDs (red droplets, marker of the quiescent phenotype) in various size in adherent cells (Figure 3I(E)). Moreover, HSCs started to produce filament fibers conferring a star-shaped morphology (Figure 3I(D)). Finally, immunofluorescence (Figure 3I(F,G)) confirmed that the isolated HSCs were positive for desmin and GFAP, both markers of the quiescent phenotype. All the above observation confirms the presence of the typical quiescent phenotype for the isolated HSCs.

#### Activation of HSCs in culture

Previous studies have reported that HSCs change their shape *in vitro* when acquiring the activated myofibroblast-like phenotype [15,17,20,40]. Our data show that following *in vitro* culturing over days 1, 3, and 7, the ‘body’ of HSCs expanded widely while the filament fiber receded, leading to progressively enlarged cells, progressing into myofibroblast cells (Figure 3II(I–K)) as shown by ORO staining (Figure 3II(L–N)) with significant (Figure 3II(R)) loss of LDs, consistent with the activated myofibroblast-like phenotype.

Our data indicate that *α-SMA* and *collagen I* (markers of HSC activation) were significantly up-regulated by days 3 and 7 of culture, whereas the mRNA level of lecithin-retinol acyltransferase (*LRAT*) was remarkably reduced (Figure 3II(S)). Parallel increase in the expression of *α-SMA* and *collagen I* over time was confirmed by immunofluorescence (Figure 3II(O–Q) )and Western blot (Figure 3II(T)).

The activated myofibroblast-like phenotype of HSCs is also characterized by increased proliferation rate [32,40]. The cell counting was determined using the standard curve (Figure 3III(U)) and the results showed that the number of cells rapidly increased over time (Figure 3III(V)). This was paralleled by the increase in the expression of *Ki67* (marker of cell proliferation) which peaked on day 3 (Figure 3III(W), *P*-value <0.05). Cell cycle analysis also indicated a higher proportion of cells in S and G_2_/M phases at day 3 compared with days 1 and 7 (Figure 3III(X), *P*-value <0.05).

### Definition of the culture conditions suitable for the maintenance of the quiescent phenotype of HSCs

#### Effect of culture medium on the proliferation of HSCs

**Figure 4:**
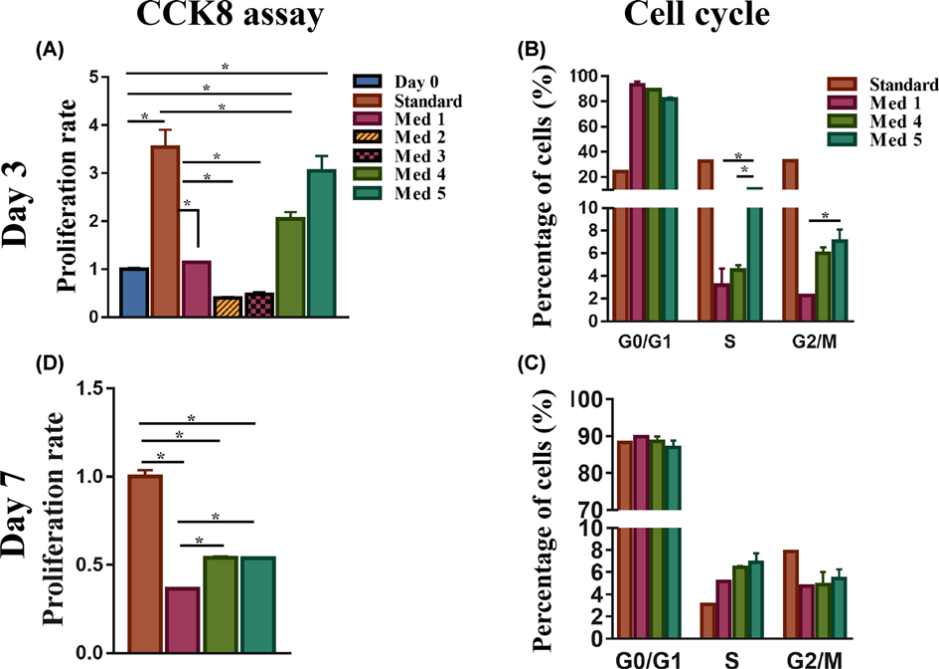
Proliferation of HSCs in different culture media HSCs cultured at day 3 (**A,B**) and day 7 (**C,D**) in standard medium and media 1–5 were evaluated for proliferation rate by the CCK8 assay and percentages of cells in the different phases of the cell cycles by cell cycle analysis (*n*=3), *P<0.05.

At day 3, the proliferation assay CCK8 showed that cells cultured in the standard medium had the highest proliferation rate (Figure 4A). Among the remaining media, all FBS-free (Table 1), HSCs in media 2 and 3 (DMEM without GLN), had the lowest cell proliferation rate (Figure 4A). Therefore, we excluded these media from further testing. Cell proliferation in medium 1, which was FBS-free, showed a decrease in proliferation compared with the standard medium (Figure 4A). Medium 4, which contained DMEM with low glucose concentration (100 mg/dl), vitA and insulin without FBS, enhanced the proliferation of HSCs compared with medium 1 (Figure 4A). However, proliferation rate was inferior compared with the standard medium. Medium 5, (DMEM with vitA and insulin but high glucose concentration (450 mg/dl)), had high proliferation rate, similarly as the standard medium (Figure 4A). At day 7, cells in media 4 and 5 displayed similar induction in proliferation, but inferior to that of the standard (Figure 4D). Moreover, media 4 and 5 kept their superiority in promoting cell growth compared with medium 1 (Figure 4D).

Cell cycle analysis performed at day 3, showed that the proportion of HSCs in S and G_2_/M phases in medium 5 was higher than in media 1 and 4 (Figure 4B, *P*-value <0.05) but lower than standard medium. At day 7, the difference in the proportion among the different medium was no longer apparent (Figure 4C). These data therefore suggest that presence of FBS and/or high glucose is conducive to HSC proliferative state. In the absence of GLN, HSC cannot proliferate.

#### Effect of culture medium on HSCs phenotype and activation

**Figure 5:**
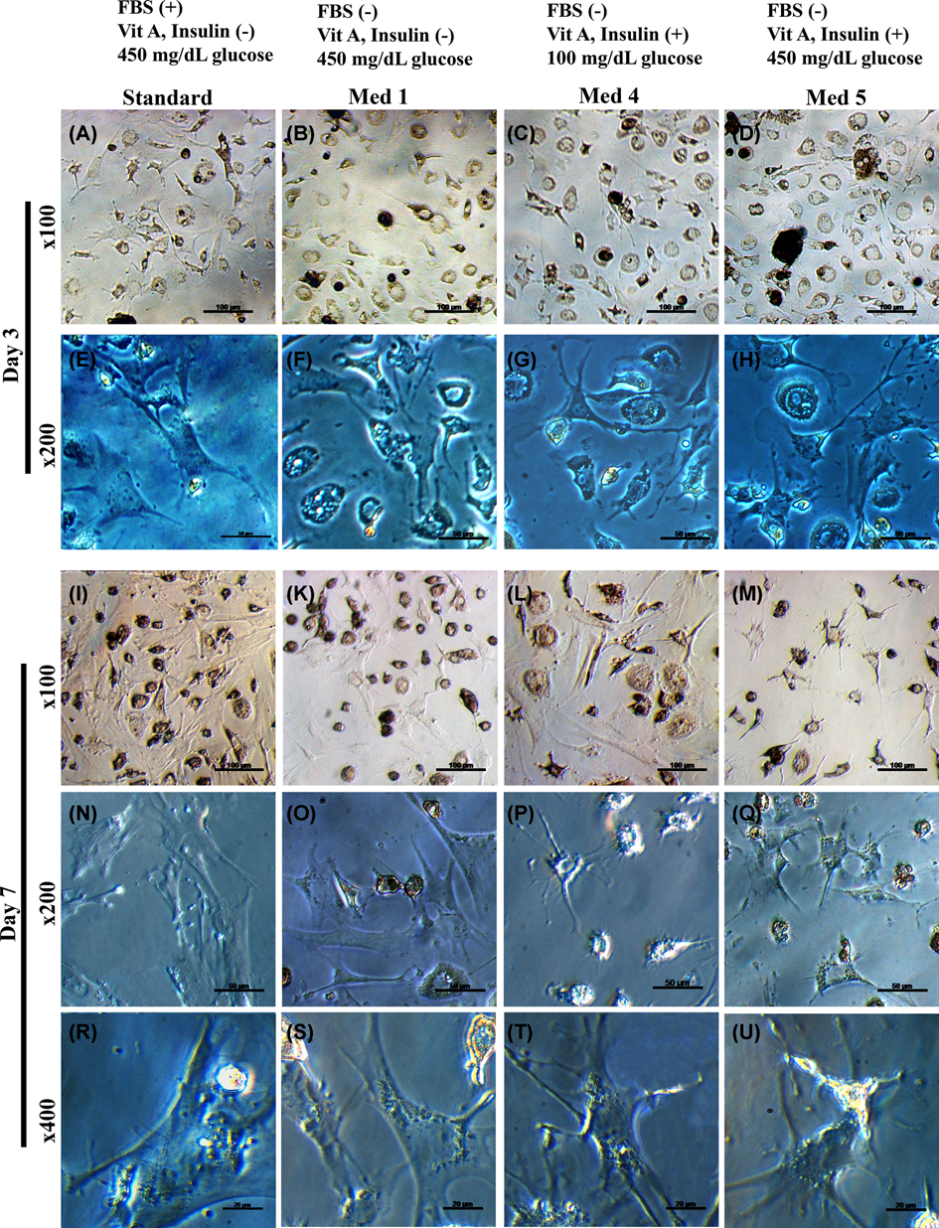
Changes in HSCs morphology by differences in culture media Representative images of the morphology by light microscope of day 3 ((**A–D**) and (**E–H**) for ×100 and ×200 magnification, respectively) and day 7 HSCs cultured ((**I–M**), (**N–Q**), and (**R–U**) for ×100, ×200, and ×400 magnification, respectively) in standard medium and media 1–5.

The bright-field images of HSCs on day 3 showed that cells in standard medium rapidly expanded their cytoplasm thus becoming well-spread compared with HSCs cultured in media 1, 4, and 5 (Figure 5). On day 7, for media 4/5, more elongated and dendritic-like filaments become evident thus indicating the acquisition of the quiescent phenotype.

**Figure 6:**
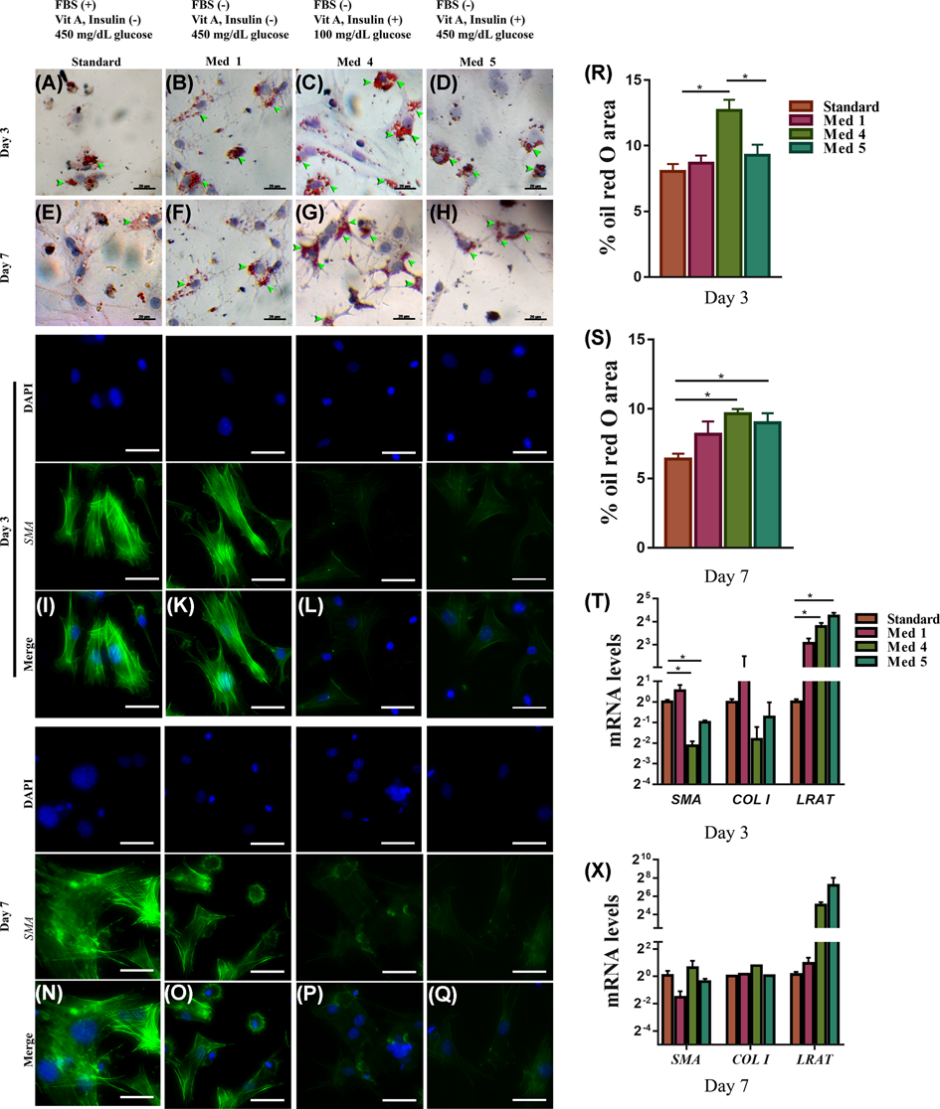
Effects of different culture media on the activation of primary HSCs Quiescent HSCs cultured under different test media (standard, 1–5) were examined for activation by LD content using ORO dye staining at day 3 (**A**–**D**) and day 7 (**E–H**); by immunofluorescent staining for α-SMA day 3, (**N–Q**) and day 7 (**I,K–M**). Quantification of LDs accumulation at day 3 (**R**), and day 7 (**S**) were shown. mRNA levels of the activation-related genes (a-SMA, COL I) and quiescent-related gene (LRAT) at day 3 (T), and day 7 (**X**). Data are shown as means ± SEM, *n*=3, *P<0.05. The arrowheads show the ORO-stained area. Scale bar: 50 μm were not noted. Abbreviation: COL I, type I collagen.

We evaluated other markers of activation, such as the loss of cytoplasmic LDs. On day 3, LD contents were highest in medium 4 compared with standard or media 1 and 5 (Figure 6A–D,R). At day 7, LDs were still detectable in media 4/5 (Figure 6E–H) and were higher in amount compared with the standard medium (Figure 6S), suggesting more HSCs in media 4 and 5 being in the quiescent state.

Consistent with the above findings, the gene expression of the pro-fibrotic genes *α-SMA* and *collagen I* was up-regulated at day 3 in HSCs cultured in medium 1 and standard medium compared with media 4 and 5 (Figure 6T). This was associated with parallel changes in the mRNA amount of *LRAT*, a marker for HSCs *in vivo* quiescence, with higher levels in HSCs maintained in media 4 and 5 compared with standard medium (Figure 6T) or medium 1 (Figure 6T) at day 3. At day 7, no major differences were seen among the different media for both *α-SMA* and *collagen I* genes (Figure 6X). Immunofluorescent staining with *α-SMA* confirmed mRNA data showing that cells cultured in medium 1 and standard medium have acquired the myofibroblastic phenotype with the presence of *α-SMA* in day 3 (Figure 6I–M) and 7 (Figure 6N–Q) compared with cells cultured in media 4 and 5.

## Discussion

Primary HSCs have become a necessary tool in drug development for the treatment of hepatic fibrosis. However, the reproducibility of current isolation procedures is inconsistent, and the maintenance of original characteristics of primary HSCs in culture is also suboptimal. While Nycodenz is the most commonly used agent in density gradient centrifugation, its concentrations vary significantly between studies, with variable efficiency of cell yield and purity. Our results suggest that Nycodenz at the 11% concentration has the best cell yield but at the expense of more cell contaminants. The first finding in our quest for the optimal isolation approach is that Nycodenz at 9.6% is optimal for HSC capture with the least contamination.

Our second finding is that HSCs isolated with 9.6% Nycodenz better retain the undifferentiated phenotype. Post-isolation HSCs are circular, ranging from 12 to 20 μm in diameter, are rich in LDs, thus perfectly resembling the HSCs isolated from previous groups [18,33,41]. They also express desmin, a widely used marker for HSCs [17,42,43].

As expected, HSCs in culture switch from the quiescent to the activated state with changes in their morphology from star-like shape to myofibroblast-like shape and progressive loss of the LDs. Their activation is characterized by the induction of the fibrogenic genes *α-SMA* and *collagen I*, and the down-regulation of the quiescent-related gene, *LRAT*. Thus, our method of HSCs isolation favors the maintenance of HSC in a quiescent state. This, in turn, allows to study their transition from the quiescent to the activated state in a simplified model of LF whose molecular mechanisms have not been fully understood.

The third finding of our investigation is related to the possibility to maintain the HSCs in a quiescent state without cell loss for a prolonged time in culture by modulating the culture medium composition. First, FBS has to be completely excluded from the culture medium as TGFβ contained in FBS may promote cell activation. Second, the optimal combination of media supplements such as glucose, GLN, insulin, and vitA previously shown to have effects on HSCs behaviors [34,35,37,40] is critical. Indeed, treatment with vitA has been reported to increase the accumulation of LDs (a feature of quiescent HSCs) and to up-regulate the GFAP (a marker for quiescent HSC) in HSCs [34]. In addition, a certain degree of proliferation is needed to allow HSC propagation in culture. High glucose concentration (450 mg/dl) promotes proliferation together with the expression of collagen in rat and human HSC [35]. To enhance the proliferation of HSC, also the supplement of l-GLN has been explored. GLN is one of the essential amino acids which plays a crucial and unique metabolic function [36]. Many studies have shown the effect of GLN on the proliferation of different cell lines *in vitro* [37–40] including HSCs. The proliferation of LX2 (an immortalized cell line of activated HSCs) and of activated rat HSC was reduced markedly in the condition of GLN depletion [40].

Although many previous studies have shown the effect of those individual elements in HSCs culture medium, the optimal combination of these elements in the culture medium has not been explored. Therefore, we hypothesize that an appropriate combination of these elements in culture could potentially keep HSCs quiescent while promoting proliferation better than a typical medium. Our data show that the medium combination (medium 4) composed of DMEM supplemented with glucose at low concentration (100 mg/dl), FBS-free, GLN (0.5 mM), vitA (100 μM), and insulin (50 ng/ml) is the best to maintain HSCs in a quiescent phenotype without losing the ability for some proliferative activity, thus allowing the cells to be perpetuated in culture. In this respect, GLN is necessary as its deprivation (such as in media 2 and 3) inhibits HSCs proliferation [40,44]. VitA and insulin have been shown to be important for the quiescent phenotype [34]. In their absence (such as in medium 1, but with GLN) HSC acquired the activated phenotype, with the loss of the LDs, the increase in the fibrogenic genes *α-SMA* and *collagen I* and the reduction in *LRAT* expression. With regard to the glucose concentration, cells cultured in the lower glucose concentration of 100 mg/dl (medium 4) better maintain their inactivated state as shown by lower lipid content and a decrease in expression of the fibrogenic markers *α-SMA* and *collagen I* compared with high glucose concentration of 450 mg/dl (medium 5).

In conclusion, Nycodenz at 9.6% is the most effective concentration to employ for HSCs isolation procedure. The optimal culture condition to maintain HSCs in the quiescent phenotype can be achieved with DMEM supplemented with glucose at low concentration (100 mg/dl), GLN (0.5 mM), vitA (100 μM), and insulin (50 ng/ml) in the absence of FBS. Under these conditions, HSC biology can be manipulated *in vitro* to further understand the mechanism of HSCs activation in hepatic fibrosis and to develop novel therapeutic strategies for this pathological condition.

## Data Availability

All data generated or analyzed during the present study are included in this published article (and its supplementary information files).

## Abbreviations

BSA: bovine serum albumin
CCK8: cell counting kit 8
DAPI: 4ʹ,6-diamidino-2-phenylindole
DMEM: Dulbecco’s modified Eagle’s medium
FBS: fetal bovine serum
*Gapdh*: glyceraldehyde-3-phosphate dehydrogenase
GFAP: glial fibrillary acidic protein
GLN: glutamine
HSC: hepatic stellate cell
ICC: immunocytochemistry
LD: lipid droplet
LF: liver fibrosis
*LRAT*: lecithin-retinol acyltransferase
ORO: Oil Red O
PFA: paraformaldehyde
qRT-PCR: quantitative reverse transcription-polymerase chain reaction
RT: room temperature
TBST: Tris-buffered saline, 0.1% Tween 20
vitA: vitamin A
α*-SMA*: α-smooth muscle actin
